# 269. A Review of Gram Negative Endogenous Endophthalmitis at University Hospital in Newark

**DOI:** 10.1093/ofid/ofab466.471

**Published:** 2021-12-04

**Authors:** Hernando Salazar, Catherine Ye, Brian Schott, Kristin R Riddle, Diana Finkel

**Affiliations:** 1 Rutgers New Jersey Medical School, Newark, New Jersey; 2 NJMS Rutgers University, Newark, NJ

## Abstract

**Background:**

Endophthalmitis (EO) is an ocular emergency characterized by intraocular inflammation, usually in response to infection. While most cases are exogenous, gram negative (GN) EO account for 10-24% of all cases, and endogenous EO (EEO) account for 2-8% of all cases. Risk factors for EEO include diabetes mellitus (DM), IV drug use, and indwelling catheters. Major sources of infection are urinary tract infections (UTI), liver abscesses, pneumonia, and bacteremia. Common pathogens include K. pneumoniae, P. aeruginosa, and H. influenzae. Outcomes are poor, with only 20% of patients achieving improved visual acuity, and 30-40% requiring enucleation.

**Methods:**

Retrospective analysis was performed on patients diagnosed with EO (n=89) at University Hospital in Newark from January 2016 to December 2020 using ICD-10 codes H44.0-H44.009, H44.1, and H44.19. Patients included were 18 years of age or older with culture proven GN endogenous EO (GNEEO) (n=7). Outcomes included anatomical success, functional success, and mortality at 28 days and 3 months.

**Results:**

7 of 89 patients met criteria for GNEEO (median age 67, 4 males, 71.4% Hispanic/Latino). Comorbidities included hepatobiliary disease (57.1%) and DM (42.9%). All 7 patients presented with ocular symptoms and 3 had non-ocular symptoms. Primary sources of infection included UTI, prostate abscess, and pneumonia/empyema. Eye cultures identified Pseudomonas in 4 patients and Klebsiella in 3 patients. Mean antibiotic length was 17.7 days with 6 patients receiving intravitreal antibiotics. Enucleation was performed in 3 patients. 2 patients had functional success and 4 had anatomical success, with 0 mortality at 28 days and 3 months.

Table 1. Ocular symptoms on presentation of cases of gram negative endogenous endophthalmitis

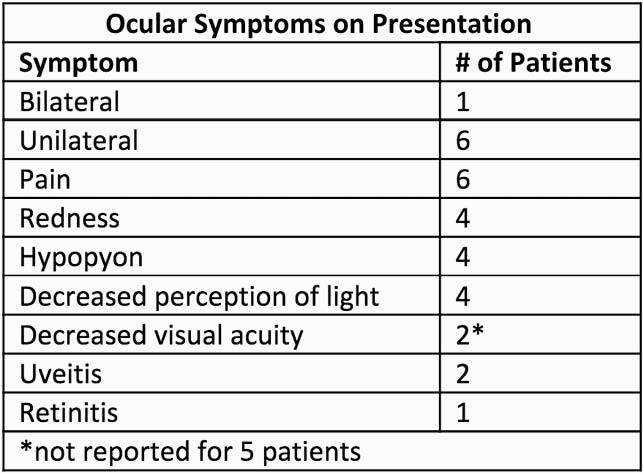

Table 2. Positives cultures obtained from cases of gram negative endogenous endophthalmitis

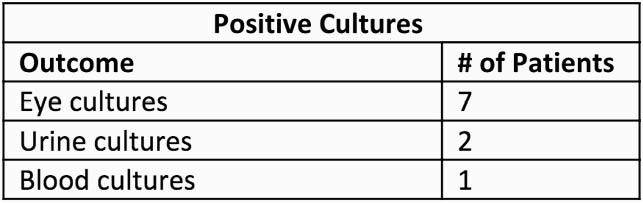

**Conclusion:**

Although rare, GNEEO causes significant morbidity, with only 2 recovering visual acuity and 3 requiring enucleation. Risk factors, sources of infection, and microbes were all consistent with those in previous reports. Hepatobiliary disease and DM were the most prominent risk factors while sources of infection included UTI and empyema. Eye cultures were positive for K. pneumoniae and P. aeruginosa, two common pathogens previously identified. This case series highlights the importance of prompt recognition and initial treatment of GNEEO with empiric coverage that includes vancomycin and ceftazidime.

**Disclosures:**

**All Authors**: No reported disclosures

